# Pathologic and Protective Roles for Microglial Subsets and Bone Marrow- and Blood-Derived Myeloid Cells in Central Nervous System Inflammation

**DOI:** 10.3389/fimmu.2015.00463

**Published:** 2015-09-08

**Authors:** Agnieszka Wlodarczyk, Oriane Cédile, Kirstine Nolling Jensen, Agathe Jasson, Jyothi Thyagabhavan Mony, Reza Khorooshi, Trevor Owens

**Affiliations:** ^1^Department of Neurobiology Research, Institute for Molecular Medicine, University of Southern Denmark, Odense, Denmark; ^2^Department of Biology, École Normale Supérieure de Lyon, Lyon, France

**Keywords:** microglia, EAE, CD11c, myeloid cells, CNS, M1/M2

## Abstract

Inflammation is a series of processes designed for eventual clearance of pathogens and repair of damaged tissue. In the context of autoimmune recognition, inflammatory processes are usually considered to be pathological. This is also true for inflammatory responses in the central nervous system (CNS). However, as in other tissues, neuroinflammation can have beneficial as well as pathological outcomes. The complex role of encephalitogenic T cells in multiple sclerosis and its animal model experimental autoimmune encephalomyelitis (EAE) may derive from heterogeneity of the myeloid cells with which these T cells interact within the CNS. Myeloid cells, including resident microglia and infiltrating bone marrow-derived cells, such as dendritic cells (DC) and monocytes/macrophages [bone marrow-derived macrophages (BMDM)], are highly heterogeneous populations that may be involved in neurotoxicity and also immunoregulation and regenerative processes. Better understanding and characterization of myeloid cell heterogeneity is essential for future development of treatments controlling inflammation and inducing neuroprotection and neuroregeneration in diseased CNS. Here, we describe and compare three populations of myeloid cells: CD11c^+^ microglia, CD11c^−^ microglia, and CD11c^+^ blood-derived cells in terms of their pathological versus protective functions in the CNS of mice with EAE. Our data show that CNS-resident microglia include functionally distinct subsets that can be distinguished by their expression of CD11c. These subsets differ in their expression of Arg-1, YM1, iNOS, IL-10, and IGF-1. Moreover, in contrast to BMDM/DC, both subsets of microglia express protective interferon-beta (IFNβ), high levels of colony-stimulating factor-1 receptor, and do not express the Th1-associated transcription factor T-bet. Taken together, our data suggest that CD11c^+^ microglia, CD11c^−^ microglia, and infiltrating BMDM/DC represent separate and distinct populations and illustrate the heterogeneity of the CNS inflammatory environment.

## Introduction

The contribution of myeloid cells to central nervous system (CNS) function is increasingly appreciated. This is especially relevant in CNS inflammation, where they can act not only as participants in the immune attack but also as regulators of the immune response and promoters of neuronal and synaptic protection and regeneration (1). A key aspect of the inflammatory response within the CNS is the recruitment and reactivation of infiltrating CD4^+^ T cells and direction of their effector function within the tissue (2). Their role in multiple sclerosis (MS) and its animal model experimental autoimmune encephalomyelitis (EAE) is complex. Although there is a consensus that inhibition or blockade of such responses has therapeutic benefit in MS and EAE [reviewed in Ref. (3)], there are numerous reports of beneficial effect of the activation of such T cells for myelin clearance, remyelination, and repair (4, 5). The cells implicated in interaction with CD4^+^ T cells within the inflamed CNS are myeloid cells, such as infiltrating dendritic cells (DC) and monocytes/macrophages, and also resident microglia (6–9). These are very heterogeneous populations of immune cells that can dampen inflammation or contribute to pathological events. Better characterization of the heterogeneity of blood-derived as well as CNS-resident myeloid cells and their interaction with T cells is crucial for understanding how to effectively control inflammation and induce neuroprotection and neuroregeneration in diseases like MS.

Microglia are cells of myeloid lineage considered to be CNS macrophages [reviewed in Ref. (10)]. However, they differ in terms of origin: while macrophages originate from fetal liver, microglia emerge from yolk sac and colonize the CNS early during neonatal development (11). Microglia are autonomously maintained through proliferation and in normal circumstances, they are not replaced by bone marrow (BM)-derived cells (12). Based on many phenotypic similarities between microglia and macrophages, the discrimination between these two populations as well as other BM- or blood-derived cells during neuroinflammation is challenging. The difference in the expression level of cell membrane tyrosine phosphatase CD45 can be used to discriminate CD45^low^ microglia from CD45^high^ blood-derived cells by flow cytometry (13, 14). In addition, other common markers used for microglial identification include CD11b, CD11c, Iba1, and CX3CR1 (10, 15–20). However, these can also be expressed by macrophages as well as monocytes and DC. Recent development of a double knock-in red (CCR2)/green (CX3CR1) mouse has allowed the discrimination of CNS-resident microglia from infiltrating leukocytes, microglia being CX3CR1-positive but, in contrast to infiltrating immune cells, CCR2-negative (21).

In this study, we aimed to examine the heterogeneity amongst microglia as well as to compare them to infiltrating blood-derived myeloid cells in EAE. We show that CD11c^+^ microglia, CD11c^−^ microglia, and CD11c^+^ blood-derived cells are functionally distinct populations of myeloid cells within the inflamed CNS. Our findings illustrate the heterogeneity of the CNS inflammatory environment, reflecting the complexity of immunological processes that lead to pathology, tissue protection, or immunoregulation.

## Materials and Methods

### Mice

Female C57BL/6j bom (B6) mice aged 6–8 weeks were obtained from Taconic Europe A/S (Lille Skensved, Denmark) and CX3CR1^gfp/gfp^ were obtained from The Jackson Laboratory (Bar Harbor, ME, USA) and maintained in the Biomedical Laboratory, University of Southern Denmark (Odense). All experiments were approved by the Danish Animal Experiments Inspectorate (approval number 2014-15-0201-00369).

### Induction of neuroinflammation

#### NMO-like disease

To induce NMO-like lesions, 7–10-week-old female B6 mice received IgG antibodies isolated from NMO patients and human complement by injection in the striatum, as described in Ref. (22). The use of human material was approved by The Committee on Biomedical Research Ethics for the Region of Southern Denmark (ref. no. S-20080142).

#### Cuprizone-induced demyelination

In order to induce demyelination, 7–12-week-old female B6 mice were fed with cuprizone for 4 weeks as described in Ref. (23).

#### EAE-model

Seven to ten weeks old female mice were immunized by injecting subcutaneously 100 μl of an emulsion containing 100 μg of myelin oligodendrocyte glycoprotein (MOG)p35–55 (TAG Copenhagen A/S, Frederiksberg, Denmark) in incomplete freunds adjuvant (DIFCO, Alberstslund, Denmark) supplemented with 400 μg H37Ra *Mycobacterium tuberculosis* (DIFCO). *Bordetella pertussis* toxin (300 ng; Sigma-Aldrich, Brøndby, Denmark) in 200 μl of PBS was injected intraperitoneally at day 0 and day 2. Animals were monitored daily from day 5 and scored on a 6-point scale as follows: 0, no symptoms; 0.5, partial loss of tail tonus; 1, complete loss of tail tonus; 2, difficulty to right, 3, paresis in one or both hind legs; 4, paralysis in one or both hind legs; 5, front limb paresis; and 6, moribund. Severe EAE usually developed 14–18 days after immunization and was defined as a score of 3–5.

### Isolation of mononuclear cells from spleen and CNS

**Figure 1:**
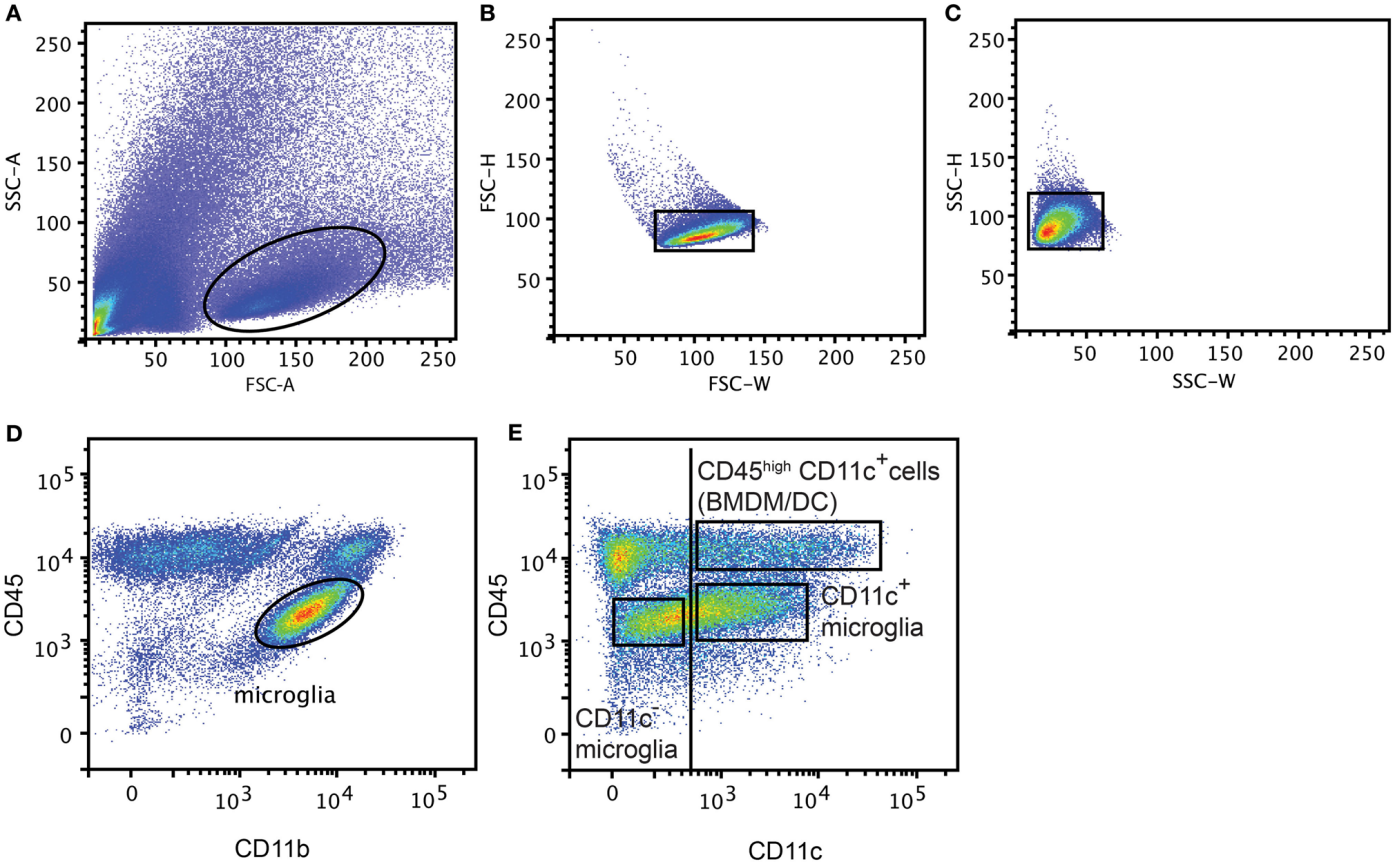
**Gating strategy for flow cytometry and FACS-sorting**. Representative flow cytometry profiles from individual central nervous system suspensions prepared from mice with severe EAE, showing gating strategy used for flow cytometry and FACS-sorting. First doublets were excluded from live gate **(A)** based on FSC-H, FSC-W **(B)** and SSC-H, SSC-W **(C)**, then microglia were gated based on CD45 and CD11b expression **(D)**. CD11c^−^ microglia, CD11c^+^ microglia, and BMDM/DC population were discriminated based on CD45 and CD11c expression and gated based on isotype control antibodies (vertical line) and sorted **(E)**.

Mice were anesthetized with 0.2 mg pentobarbital (200 mg/ml; Glostrup Apotek, Glostrup, Denmark) per gram body weight and intracardially perfused with ice-cold PBS. CNS tissue (Brain and SC from EAE model, Ipsi lateral part of the brain from NMO-like disease, and whole brain from Cuprizone-induced demyelination) was collected and a single cell suspension was generated by forcing through a 70 μm cell strainer (BD Biosciences). Mononuclear cells were collected after centrifugation on 37% Percoll (GE Healthcare Bio-sciences AB, Brøndby, Denmark). Spleens were digested with collagenase D (0.5 mg/ml; Roche, Hvidovre-Copenhagen) and DNase I (40 μg/ml, Roche) for 30 min at 37°C. Supernatant was collected and supplemented with 100 mM EDTA for 5 min at 37°C and passed through a 70 μm cell strainer (BD Biosciences, Albertslund, Denmark). Red blood cells were lysed with a 0.83% NH_4_Cl solution. Splenocytes and CNS mononuclear cells were then incubated with anti-Fc receptor (Clone 2.4G2; 1 μg/ml; BD Pharmingen) and Syrian hamster IgG (50 μg/ml; Jackson Immuno Research Laboratories Inc., Skanderborg, Denmark) in PBS 2% fetal bovine serum (FBS). CNS mononuclear cells were stained with PE-anti-mouse CD45 (Biolegend, Copenhagen, Denmark), PerCP-Cy5.5-anti-mouse CD11b (Biolegend) and biotinylated-anti-mouse CD11c (BD Pharmingen) antibodies in PBS 2% FBS. Splenocytes were stained with PE-anti-mouse CD11c antibodies in PBS 2% FBS and CD11c^+^ cells were sorted on a FACSAria™ III cell sorter (BD Biosciences, Albertslund, Denmark). After excluding doublets (FSC-H, FSC-W and SSC-H, SSC-W) (Figures 1A–C), CNS cell populations were gated based on isotype control antibodies as CD45^dim^CD11b^+^CD11c^−^ (CD11c^−^ microglia), CD45^dim^CD11b^+^CD11c^+^ (CD11c^+^ microglia), and CD45^high^CD11c^+^ (BMDM/DC) (Figures 1D,E) and were sorted on a FACSAria™ III cell sorter (BD Biosciences).

### Bromodeoxyuridine (BrdU) proliferation assay

B6 mice received 100 μl of 1 mg/ml BrdU by daily intraperitoneal injection, starting from day 10 after immunization. Mice with severe EAE were sacrificed and analyzed by flow cytometry using BrdU flow kit (BD Pharmingen) according to the manufacturer’s protocol. Data were acquired on an LSRII™ flow cytometer and analyzed using FACSDiva™ 7 software (BD Biosciences).

### RNA extraction, reverse transcription, and quantitative real-time PCR

Sorted splenic CD11c^+^ cells from individual mice as well as CD11c^+^ microglia, CD11c^−^ microglia, and CD45^high^CD11c^+^ cells from pools of 2–3 mice were placed in RLT buffer (Qiagen, Copenhagen, Denmark). Total RNA was extracted using RNeasy columns as per the manufacturer’s protocol (Qiagen). Reverse transcription was performed with M-MLV reverse transcriptase (Invitrogen) according to the manufacturer’s protocol.

Quantitative real-time PCR (qPCR) was performed with 1 μl cDNA in a 25 μl reaction volume containing Maxima^®^ Probe/ROX qPCR Master mix (Fermentas, St. Leon-rot, Germany), forward and reverse primers (800 nM; from TAG Copenhagen A/S, Frederiksberg, Denmark) and probe (200 nM; Applied Biosystems, Nærum, Denmark and TAG Copenhagen A/S, Frederiksberg, Denmark). Primer and probe sequences were as follows: IFNβ: *For*: GCGTTCCTGCTGTGCTTCTC; *Rev*: TTGAAGTCCGCCCTGTAGGT; *Probe*: CGGAAATGTCAGGAGCT; IRF7: *For*: CACCCCCATCTTCGACTTCA; *Rev*: CCAAAACCCAGGTAGATGGTGTA; *Probe*: CACTTTCTTCCGAGAACT; IFR3: *For*: CACCCCAAGAAAATCCACTGA; *Rev*: AGGCGGTCACCTCGAACTC; *Probe*: TAGCTGAGGAACAATG. TaqMan^®^ PreAmp Master Mix Kits were used for T-bet: Mm01299452; YM1: Mm00657889; ARG1: Mm00475988; iNOS: Mm00440502; CSF1R: Mm01266652. For IGF1, SYBR Green/ROX qPCR Master Mix (2X) Probe/ROX qPCR Master mix (Fermentas, St. Leon-rot, Germany) was used with forward and reverse primers (800 nM) from TAG Copenhagen A/S). Primer sequences were as follows: IGF1: *For*: CCG AGG GGC TTT TAC TTC AAC AA; *Rev*: CGG AAG CAA CAC TCA TCC ACA A. PCR reactions were done on an ABI Prism 7300 Sequence Detection System (Applied Biosystems). Results were expressed relative to 18S rRNA (2ΔCT method) as endogenous control (TaqMan^®^ Ribosomal RNA control Reagents kit; Applied Biosystems). cDNA was diluted 1/500 for 18S rRNA analysis.

### Histology

Fifty-micrometer sections (coronal and sagittal) from 4% PFA-fixed, frozen brains of PBS-perfused B6 mice were cut on a cryostat and stored in de Olmos cryoprotectant solution (24) at −14°C. Sections were washed in PBS and incubated for 30 min with 10% methanol and 10% H_2_O_2_ in PBS to block endogenous peroxidase. Alternatively 12 μm spinal cord sections were fixed with 4% PFA. After repeated rinses with 0.2% Triton X100 in PBS (PBST), sections were incubated for 1 h in 3% BSA in PBS to block unspecific binding. Next, sections were incubated overnight at 4°C with corresponding primary antibodies: rabbit anti-Iba1 (Wako, Neuss, Germany), hamster anti-CD11c (25) (kindly provided by Dr. Diego Gomez-Nicola; Centre for Biological Sciences, University of Southampton, SO16 6YD, Southampton, UK), rat anti-mouse Mac-1/CD11b (clone MCA711, AbD Serotec, Kidlington, UK), and rabbit anti-T-bet (H-210, Santa Cruz Biotechnology). Following primary antibody incubation, the sections were washed with PBST and incubated for 1 h with the appropriate secondary antibody: goat anti-Armenian hamster IgG-HRP (Santa Cruz Biotechnology, Heidelberg, Germany), goat anti-rabbit Alexa 488 (Invitrogen, Taastrup, Denmark), goat anti-rat Alexa 488 or biotinylated goat anti-rabbit (Abcam, Cambridge, UK) and PE-streptavidin (Biolegend). To visualize CD11c following secondary antibody incubation, the sections were washed with PBS and incubated for 4 min with TSA™ Plus Cy3 System (PerkinElmer, Skovlunde, Denmark). After immunofluorescent labeling, the sections were counterstained with DAPI and mounted with Fluorescence Mounting Medium (DAKO, Glostrup, Denmark). The sections were visualized on an Olympus FV1000MPE Confocal microscope and analyzed by FV10-ASW 4.06 software (Olympus, Denmark).

### Statistical analysis

All experiments were repeated at least three times and data are presented as means ± SEM. Statistical significance was assessed using the two-tailed Mann–Whitney *U*-test (GraphPad Prism 4). *P*-values <0.05 were considered significant.

## Results and Discussion

### CD11c^+^ microglia emerge during neuroinflammation

**Figure 2:**
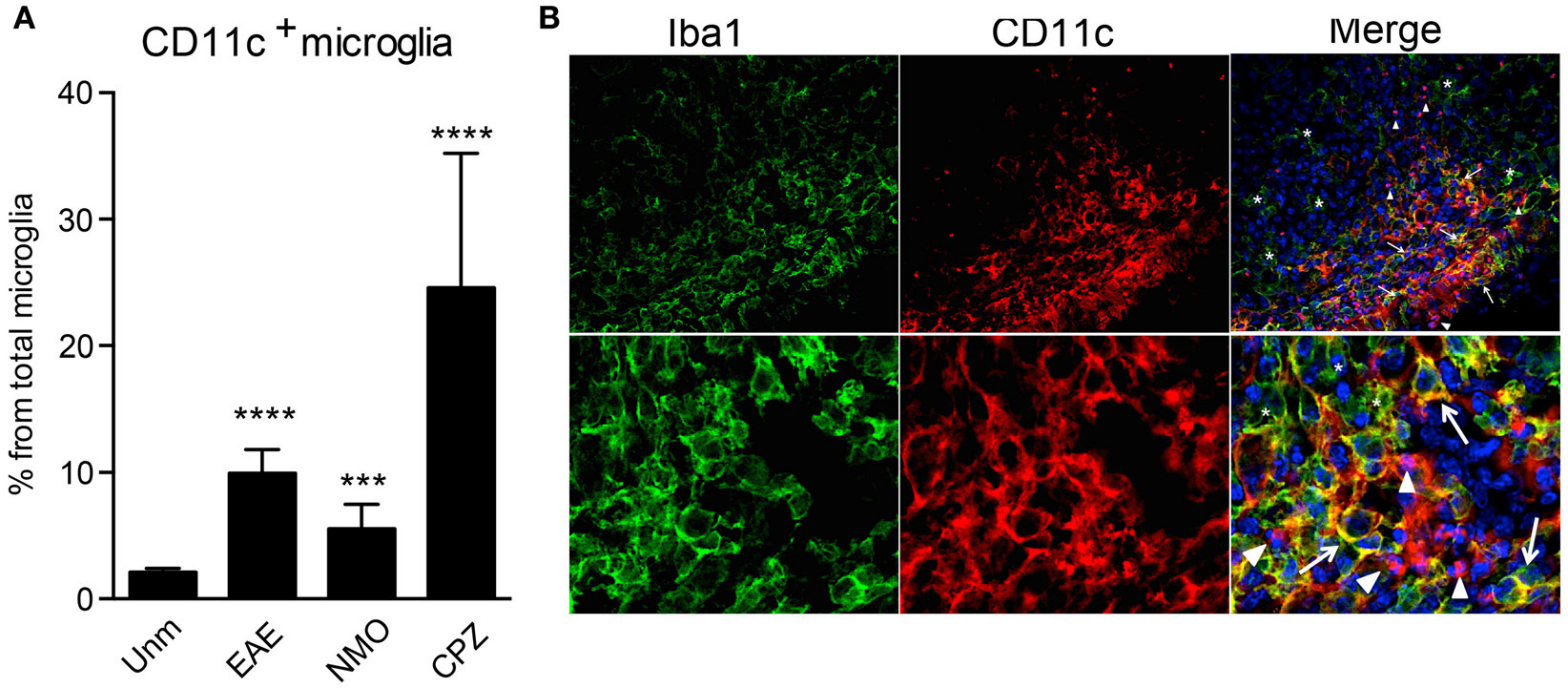
**CD11c^+^ microglia emerge in response to neuroinflammation**. **(A)** Flow cytometry analysis shows a significant increase of CD11c^+^ microglia, presented as a percentage of total microglia, in EAE, NMO-like disease, and cuprizone-induced demyelination. Data are presented as means ± SEM of three individual experiments (*n* ≥ 6), **P < 0.01 ***P < 0.005, ****P < 0.001. **(B)** Representative confocal microscopic analysis of spinal cord from mice with severe EAE from three individual experiments. Arrowheads point to CD11c (red) single positive cells, asterisks point to Iba1 (green) single positive cells, and arrows point to cells co-expressing CD11c marker with Iba1.

CD11b and CD11c form integrin heterodimers with CD18 and function as complement receptors. Both can be expressed by microglia [reviewed in Ref. (10)]. However, CD11c expression is usually attributed to activation of these cells. In humans, CD11c was shown to be constitutively expressed at a low level in microglia and upregulated in Alzheimer’s disease (AD) (26). CD11c^+^ Iba1^+^ cells were found in developing mouse brain as early as E16, they were also evident during postnatal neurodevelopment P2 (27), while in adult brain they were hardly visible (20, 27). We have confirmed prominence of CD11c-positive cells in the developing brain, most of them being CCR2^−^ CX3CR1^+^ Iba1^+^ microglia (unpublished data). In adult mice, only very few CD11c^+^ cells are present in the steady state, averaging 2–3% of total microglia (Figure 2A). However, their proportions increase dramatically during active neuroinflammation, such as in EAE (19), cuprizone-induced demyelination (23), and in a mouse model for neuromyelitis optica (Figure 2A), and also in an animal model for AD (28). Due to many phenotypic overlaps, bone marrow-derived macrophages (BMDM)/DC and microglia within the inflamed CNS are difficult to distinguish from each other by histology. Nevertheless, we assessed the localization of CD11c^+^ cells within the inflamed spinal cord. We identified three different populations of myeloid cells within the lesion: Iba1^+^ CD11c^+^ and Iba1^+^CD11c^−^ cells, that are likely to include microglia/BMDM, as well as CD11c^+^ Iba1^−^ cells, most likely DC. Beside differential expression of these markers, they show different morphology, ranging from round, as well as dendritic-like CD11c^+^ Iba1^−^ cells to amoeboid double-positive and Iba1-single positive cells. All of them were uniformly distributed within the lesion, with obvious possibility for interaction with T cells and with each other (Figure 2B). These data emphasize the high morphological and phenotypic heterogeneity of myeloid cell populations in neuroinflammation.

### CX3CR1 deficiency influences EAE susceptibility

**Figure 3:**
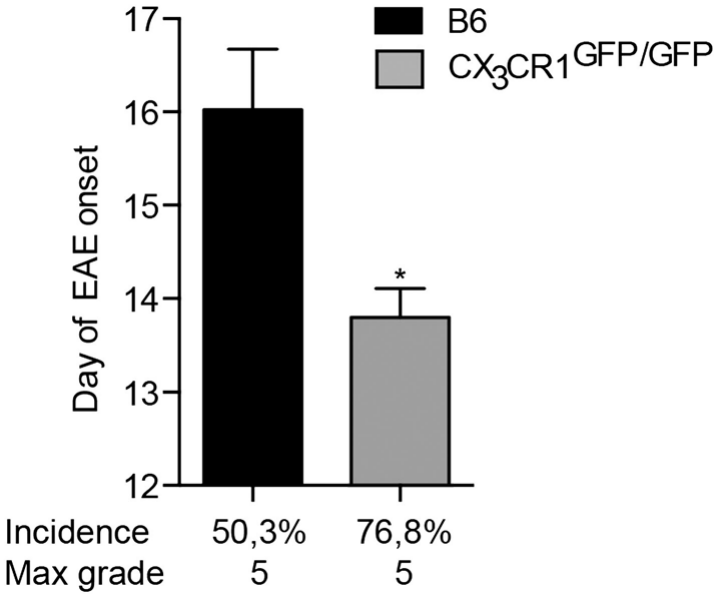
**CX3CR1-deficient mice are more susceptible to EAE**. CX3CR1^gfp/gfp^ and B6 mice were immunized with (MOG)p35–55 and monitored daily for development of EAE. The data shows significantly earlier onset, higher incidence of EAE in CX3CR1-deficient mice than in B6 mice. Data are presented as means ± SEM of at least four individual experiments (*n* ≥ 4), *P < 0.05.

Fractalkine receptor CX3CR1 and its ligand CX3CL1 are expressed within the CNS by microglia and neurons, respectively (29). Their interaction plays an important role in neurodevelopment and has also been implicated in neuroinflammation. Disruption of CX3CR1–CX3CL1 axis leads to microglial activation and hypersensitivity and may result in neurotoxicity [(18) and reviewed in Ref. (30)]. For instance, mice deficient in CX3CR1 have been reported to develop more severe EAE symptoms than WT animals (31). Here, we show that mice lacking CX3CR1 are more susceptible to EAE, with a significantly earlier onset and higher incidence of the disease (Figure 3). This data support conclusions from BM chimeras (31) that CX3CR1 regulates myeloid cell and possibly microglial responses, which in this case would act to dampen neuroinflammation, as also evidenced in CX3CR1-deficient mice (18).

### M1/M2 gene expression is not altered in CX3CR1-deficient microglia

**Figure 4:**
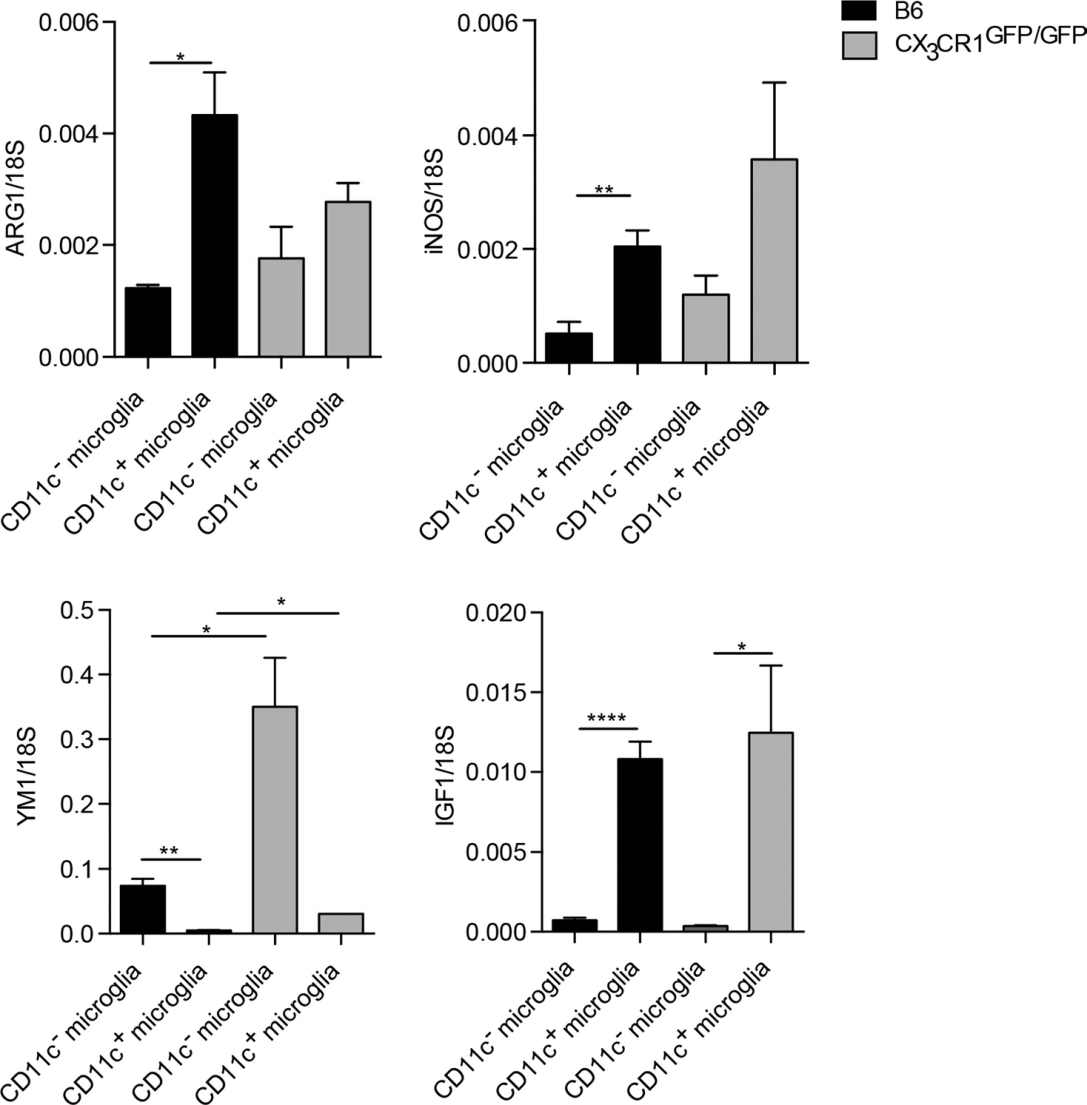
**Function-related gene expression by CD11c^−^ and CD11c^+^ microglia in CX3CR1^gfp/gfp^ and B6 mice in EAE**. Expression of IL-10, ARG1, YM1, iNOS, and IGF-1 in fluorescence-activated cell sorted CD11c^+^ microglia and CD11c^−^ microglia from the central nervous system from mice with severe EAE were analyzed by quantitative real-time PCR. Data are presented as means ± SEM of three individual experiments (*n* ≥ 5, where *n* represents a pool of 2–3 individual mice); *P < 0.05; **P < 0.01.

Microglial phenotypic heterogeneity may also reflect functional status (1, 32), as has been described for macrophages [reviewed in Ref. (33)]. Classically activated macrophages secrete inflammatory cytokines and mediators involved in host defense, such as tumor necrosis factor (TNFα), IL-12, or nitric oxide (NO) produced by inducible NO synthase (iNOS). By contrast, alternatively activated macrophages that are involved in the regulation of the immune response to limit inflammation and promote tissue repair express arginase-1 (ARG1) or chitinase-like proteins, such as YM1. Such cells also secrete growth factors, such as insulin-like growth factor 1 (IGF-1) or anti-inflammatory cytokine IL-10 (33). We have addressed functional correlates to CD11c phenotypic distinction for microglia sorted from the CNS of B6 and CX3CR1^GFP/GFP^ mice with EAE, by analysis of mRNA levels of ARG1, YM1, iNOS, IL-10, and IGF1. All of them showed clear-cut preferential expression by one or another microglial subset (Figure 4). There was no difference in TNFα expression between these microglial populations (not shown). These results, therefore, point to functional distinctions between CD11c^+^ and CD11c^−^ microglia. Moreover, CX3CR1 deficiency did not influence M1/M2-associated gene expression in microglia except for YM1, which was upregulated in CX3CR1-deficient microglia (Figure 4). These data suggest that the susceptibility to EAE in CX3CR1-deficient mice is not dependent on microglial M1/M2 phenotype.

### Myeloid cell heterogeneity in inflamed CNS in EAE

**Figure 5:**
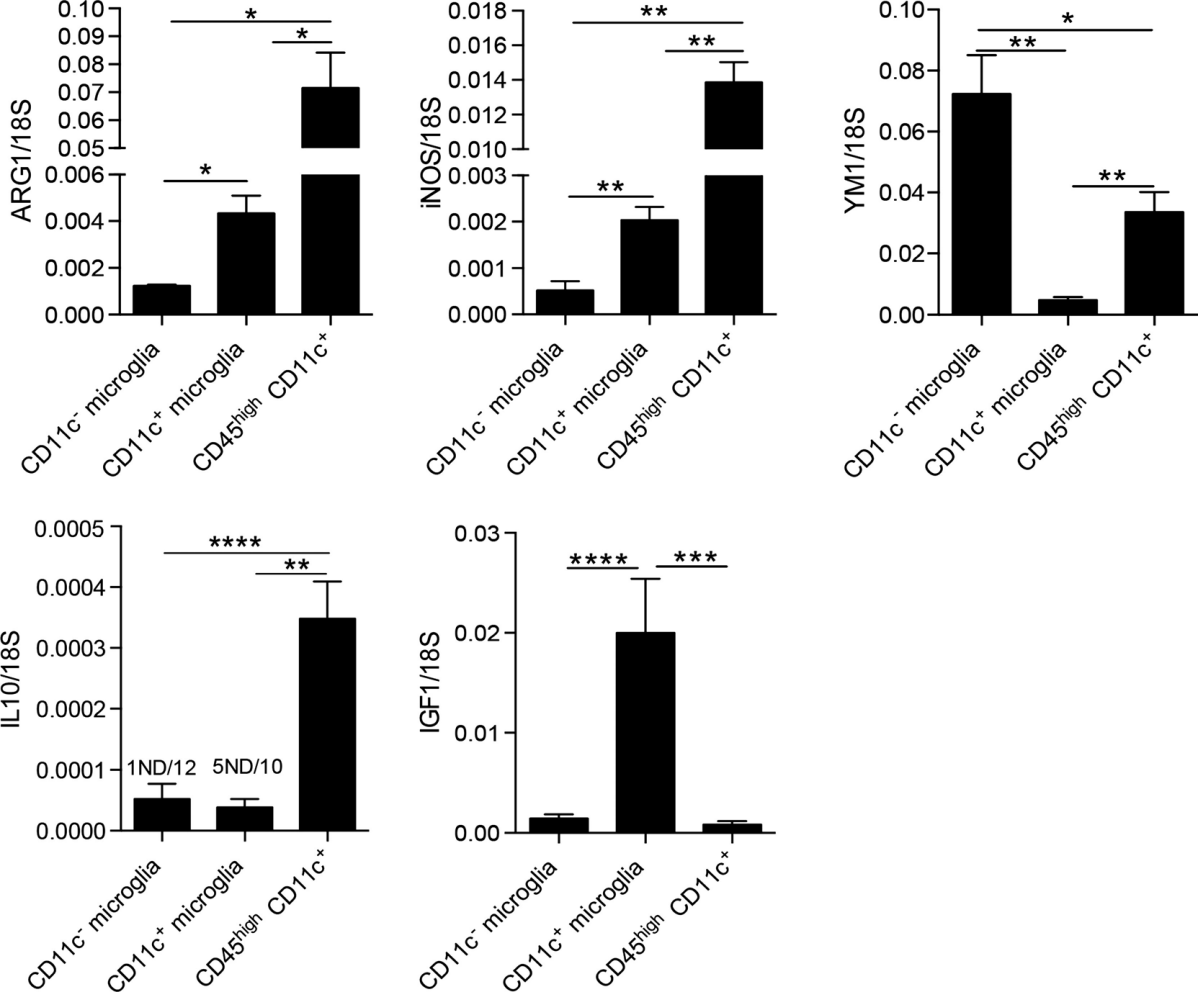
**Comparison of expression of M1/M2-associated genes in myeloid cells in CNS in EAE**. Expression of ARG1, YM1, iNOS, IL-10, and IGF-1 in fluorescence-activated cell sorted CD11c^+^ microglia and CD11c^−^ microglia and CD45^high^CD11c^+^ infiltrating cells (BMDM/DC) from the central nervous system from mice with severe EAE were analyzed by quantitative real-time PCR. Data are presented as means ± SEM of three individual experiments (*n* ≥ 5, where *n* represents a pool of 2–3 individual mice); *P < 0.05; **P < 0.01.

We were further interested in differences between microglia and blood-derived myeloid cells; thus, we compared mRNA levels of ARG1, YM1, iNOS, IL-10, and IGF1 between microglia populations and CD45^high^CD11c^+^ cells (BMDM/DC). Here, we show that BMDM/DC express significantly higher levels of ARG1, iNOS, and IL-10 than both populations of microglia, they also expressed significantly more transcripts of YM1 than CD11c^+^ microglia, but less than CD11c^−^ microglia (Figure 5). Interestingly, while CD11c^+^ microglia showed high expression of IGF1 transcripts, neither CD11c^−^ microglia nor infiltrating CD45^high^ cells expressed significant levels of this growth factor (Figure 5). Association of IGF1 expression with CD11c^+^ microglia has also been reported in glatiramer acetate-treated transgenic mice with an AD-like phenotype and has been suggested as a mechanistic basis for the ability of CD11c^+^ microglia to promote neurogenesis (34).

**Figure 6:**
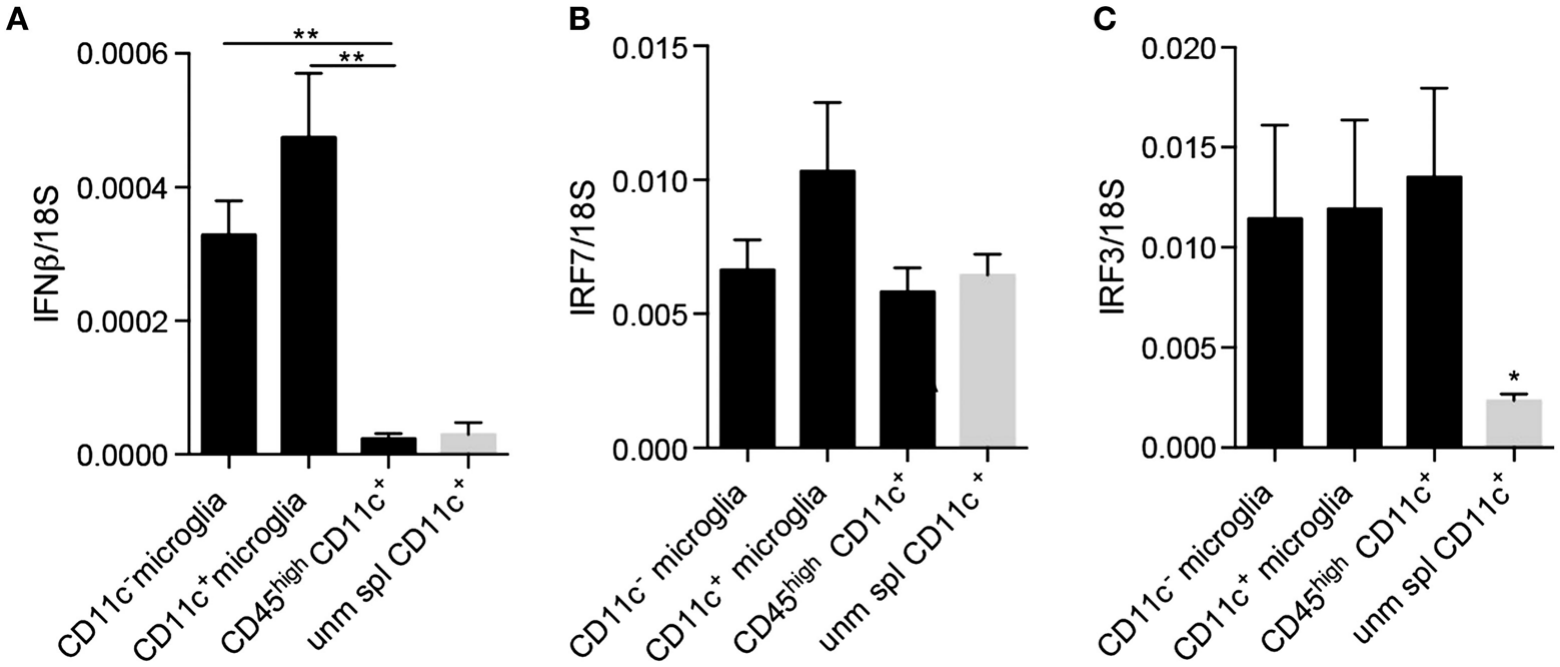
**IFNβ and IFN regulatory factors expression by microglia and infiltrating BMDM/DC in EAE**. Expression of IFNβ **(A)**, IRF7 **(B)**, and IRF3 **(C)** by sorted myeloid cells (CD11c^+^ microglia, CD11c^−^ microglia and CD45^high^CD11c^+^ BMDM/DC) from the CNS of mice with severe EAE as well as unmanipulated splenic CD11c^+^ cells was analyzed by RT-qPCR. Data are presented as means ± SEM of three individual experiments (*n* ≥ 5, where *n* represents a pool of 2–3 individual mice) **P < 0.01.

Since neurogenesis may be considered as a part of the anti-inflammatory or protective spectrum, we asked whether these cells additionally selectively expressed other regulatory cytokines. It was recently published that IFNβ produced by a subpopulation of microglia at the peak of EAE facilitated clearance of myelin debris, thus, contributing to possible explanations for amelioration of EAE as well as MS by IFNβ (35). Moreover, we have shown that experimental induction of microglial-derived IFNβ was protective in EAE (36). We, therefore, asked whether CD11c^+^ microglia expressed IFNβ in EAE. Contrary to our expectations, there was no difference in expression of IFNβ mRNA by CD11c^+^ and CD11c^−^ microglia isolated from the CNS of mice with EAE (Figure 6A). On this basis, there was, therefore, no evidence for a selective regulatory role for CD11c^+^ microglia. By contrast, infiltrating BMDM/DC expressed very low levels of IFNβ mRNA comparable to unmanipulated splenic CD11c^+^ cells, consistent with lack of a regulatory role for these cells, distinct from CNS-resident microglia. However, all of these brain myeloid populations may respond to type I IFN, as evidenced by equivalent expression of IRF7 and IRF3 (Figures 6B,C). Interestingly, IRF3 was upregulated in BMDM/DC compared to unmanipulated splenic CD11c^+^ cells (Figure 6C), suggesting ongoing active response within the CNS. These data further suggest a beneficial role for microglia in demyelinating diseases as well as emphasize functional distinctions between them and infiltrating BMDM/DC. Peripherally administered IFNβ is used as a first-line therapy for MS (37). IFNβ, constitutively expressed by microglial cells, is increased in EAE and play a protective role in the CNS (35, 36). Whether this protection might involve IFNβ-driven DC response may be speculated but would be consistent with the expression of the IFNβ response gene IRF7 by DC, which we have shown.

Our findings support observations made by Yamasaki et al. who used a combination of differential reporter gene expression and morphological correlates in EAE to infer that whereas BMDM were implicated in myelin stripping at nodes of Ranvier, microglia were not intimately associated with the demyelinating axon. Their location, relative morphology, and gene expression profile pointed to microglial involvement in phagocytosis and clearance of debris (38). Together with our findings, now based on consensus phenotypic distinction between microglia and BMDM, microglia can be seen to play a beneficial role in CNS inflammation, as has been discussed elsewhere (39).

### Myeloid cells turn-over during EAE

**Figure 7:**
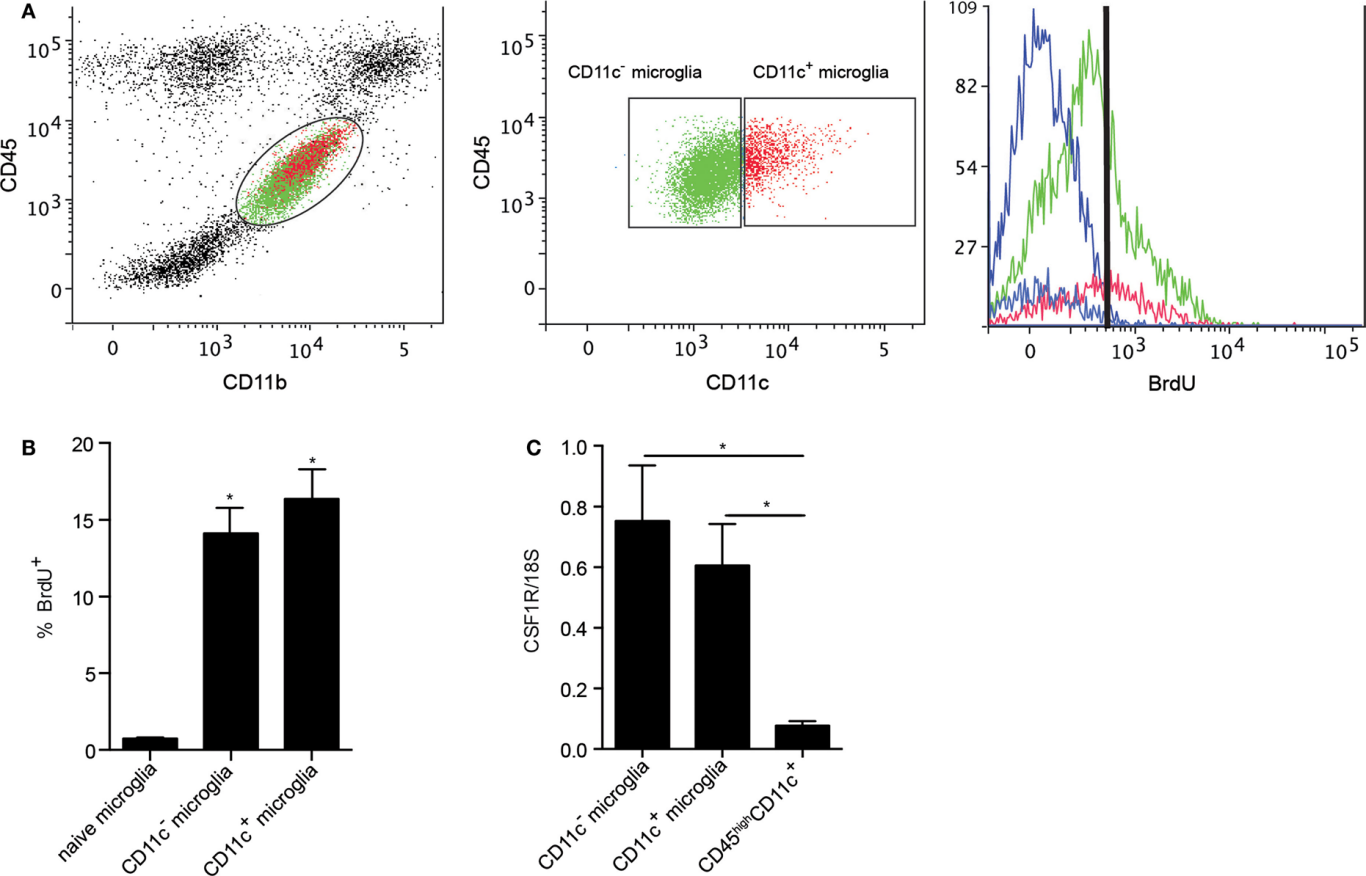
**Proliferation of microglial and BMDM/DC populations during EAE**. Representative flow cytometry profiles of six individual central nervous system suspensions prepared from mice with severe EAE showing BrdU incorporation by CD11c^−^ microglia (green) and CD11c^+^ microglia (red) based on negative control (blue) **(A)**. Flow cytometry analysis shows a significant increase in percentage of proliferating (incorporating BrdU) microglia during EAE compared to naive controls **(B)**. Expression of CSF1R in fluorescence-activated cell sorted myeloid cells (CD11c^+^ microglia, CD11c^−^ microglia, and CD45^high^CD11c^+^) from the central nervous system was analyzed by quantitative real-time PCR **(C)**. Data are presented as means ± SEM of three individual experiments (*n* ≥ 5, where *n* represents a pool of 2–3 individual mice) *P < 0.05.

Microglia have been shown to be a self-renewing population within the CNS. Their proliferation in response to neurodegeneration and their maintenance in adult mice were reported to be dependent on macrophage colony-stimulating factor receptor (CSF1R) signaling (25, 40). CSF1R signaling in response to its ligands, CSF1 and IL-34, induces microglial proliferation (40), suggesting tonic self-renewal as a component of normal homeostasis. Given the emergence of CD11c^+^ microglia in neuroinflammation, we asked whether CSF1R signaling might play a selective role in expansion of these cells. We used BrdU incorporation to assess microglial proliferation. Both CD11c^+^ and CD11c^−^ populations of microglia proliferated equivalently during EAE (Figures 7A,B), arguing against proliferation *per se* as basis for relative expansion of CD11c^+^ cells. Consistent with this, there was no significant difference in expression of CSF1R by CD11c^+^ and CD11c^−^ microglia (Figure 7C). Strikingly, infiltrating BMDM/DC (CD45^high^CD11c^+^ cells) expressed very low levels of CSF1R (Figure 7C). This again emphasizes that differences between subsets of CNS-resident microglia are minor when both are compared to BMDM/DC.

### BMDM/DC but not microglia express T-bet during EAE

**Figure 8:**
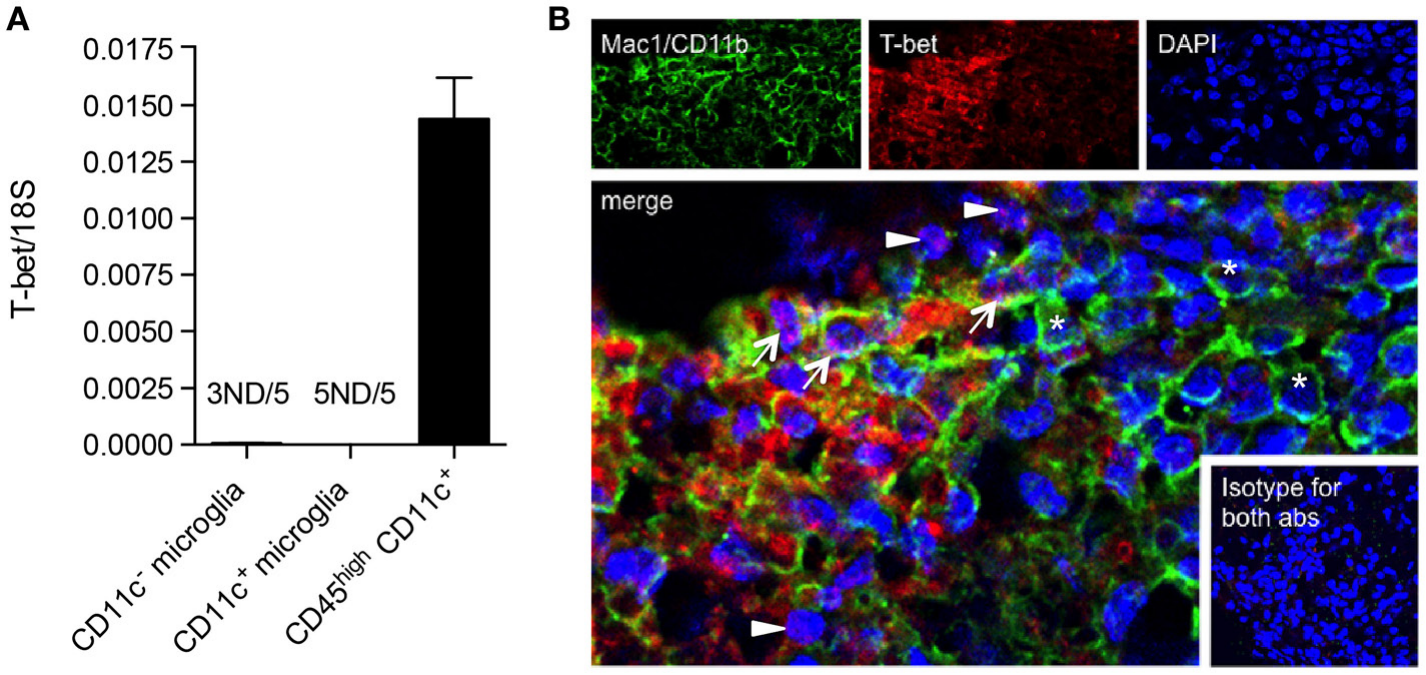
**T-bet is expressed by infiltrating BMDM/DC but not by microglia in EAE**. **(A)** Expression of T-bet in sorted myeloid cells (CD11c^+^ microglia, CD11c^−^ microglia, and CD45^high^CD11c^+^) from the central nervous system from mice with severe EAE was analyzed by quantitative real-time PCR. Data are presented as means ± SEM of three individual experiments (*n* ≥ 5, where *n* represents a pool of 2–3 individual mice). ND, not detected. **(B)** Representative confocal microscopic analysis of spinal cord from mice with severe EAE for four individual experiments. Arrowheads point to T-bet (red) single positive cells, asterisks point to Mac1/CD11b (green) single positive cells, and arrows point to cells co-expressing T-bet marker with Mac1/CD11b.

We have shown that CD11c^+^ microglia in EAE express MHC-II and co-stimulatory molecules CD80 and CD86, and that these cells have potent ability to induce proliferation of antigen-primed CD4^+^ T cells (19). This has led to speculation that they may reflect a more DC-like subset of microglia, as was previously suggested, largely on the basis of morphology and CD11c expression (41). However, they differ from DC in their relative expression of Th1- and Th17-inducing cytokines and in their quantitative ability to induce such T cell responses, CD11c^+^ microglia being, noticeably, less effective (19). We have now further explored possible overlap between CD11c^+^ microglia and DC by comparison of their expression of the transcription factor T-bet. T-bet was first isolated as a novel Th1-specific T box transcription factor that controls the expression of the hallmark Th1 cytokine, IFNγ (42). T-bet is also expressed by other IFNγ-producing cells including CD8^+^ T cells and NK cells, and has been shown to be expressed by IgG2a^+^ memory B cells, where it plays a role in IFNγ-mediated class switching as well as controlling expression of the chemokine receptor CXCR3 [reviewed in Ref. (43)]. T-bet is classically associated with the Th1 CD4^+^ T cell subset but also controls IL23R expression by Th17 (44). It has been implicated in regulation of effector responses by CD8^+^ T cells (45, 46) and in control of Th-1 expression of granulocyte–macrophage colony-stimulating factor (GM-CSF or CSF2) (47), which has been proposed to be the link between pathogenic T cells and infiltrating myeloid cells (48). T-bet was reported to be required for Th1 and Th17 encephalitogenicity (49), although this has been contradicted by later studies (50). Earlier studies had shown that T-bet was also expressed by DC and regulated DC functions (45, 46), and although reported to play a role in IFNγ production by DC, that is unlikely to be a major activity of this cell type. Expression of T-bet by DC was critical for Th1 induction and for the generation of T cell memory (43). T-bet was later shown to play a role in DC control of mast cell precursor migration (51). We compared levels of T-bet mRNA in microglia and BMDM/DC isolated from the CNS of mice with EAE. Whereas CD45^high^CD11c^+^ BMDM/DC robustly expressed high levels of T-bet mRNA, it was undetectable in most microglial samples, regardless of CD11c expression (Figure 8A). The expression of T-bet is, therefore, a selective property of infiltrating cells, likely to be specific for DC based on previous reports, and does not occur in CNS-resident microglia.

Glimcher and colleagues showed that DC express T-bet (45). Microglial expression had not previously been reported and our findings, somewhat surprisingly given the many overlaps in myeloid cell phenotype and their involvement in Th1 induction and chemokine responses, indicate that, in fact, these brain macrophages do not express significant levels of T-bet. Thus, it was of interest that such a high proportion of T-bet-expressing cells in EAE infiltrates should turn out to be DC rather than T cells.

We were interested whether T-bet^+^ DC co-localized with microglial cells within EAE lesions. Using immunohistochemistry and immunofluorescence protocols, we showed that although some CD4^+^ cells co-stained with T-bet, as expected, the majority of T-bet^+^ cells did not express CD4 (data not shown), but stained for Mac-1/CD11b (Figure 8B). These represent the sorted CD45^high^ BMDM/DC, which we showed to be the only myeloid cell source of T-bet mRNA (Figure 8A). EAE lesions are, therefore, highly heterogeneous, containing regulatory microglia with differential ability to present antigen to and induce cytokine responses from infiltrating T cells as well as infiltrating BMDM/DC that show no regulatory phenotype, many of which express T-bet.

All together, these data suggest that T-bet may be useful as a potential marker (along with myeloid markers, such as CD11b) for infiltrating DC in neuroinflammation, given that these populations of myeloid cells are morphologically indistinguishable.

### Summary

We, thus, show that CNS-resident microglia that are maintained at least in part by proliferation include functionally distinct subsets that can be distinguished by expression of CD11c. These subsets differ in their expression of ARG1, YM1, iNOS, IL-10, and IGF1. These markers have been used as a basis for a M1/M2 phenotype classification for macrophages and were extended to describe microglia. Although both subpopulations of microglia clearly differed from each other, they did not show a pure M1 or M2 phenotype. This further supports the detailed transcriptional analysis of LPS-activated macrophages, which has revealed limits to such a binary distinction (52) and is in line with other studies where microglia have been shown to express M1 and M2-related genes simultaneously (53, 54). It is, thus, probably more useful to define phenotypic/functional correlates on a case-by-case basis as, for instance, in Ref. (55). The issue of M1/M2 microglial phenotype and its limitations have been thoroughly discussed in Ref. (39).

We further show that despite these phenotypic and functional distinctions, microglia differ from blood-derived or BMDM/DC in that they express the regulatory cytokine IFNβ, to which BMDM/DC may respond but do not express, and that BMDM/DC express the Th1-associated transcription factor T-bet, whereas microglia do not. The fact that DC and microglia co-localize within inflammatory lesions in EAE emphasizes that the microenvironment in which autoreactive T cells are activated is complex and includes both regulatory and pro-pathologic myeloid cells that themselves may exert effector functions in the inflamed CNS.

## Conclusion

Over two decades of research on CD4^+^ T cell subsets based on their cytokine production profile has led to major increases in understanding of the inflammatory process. Myeloid cells in neuroinflammation also show heterogeneity and may need to be equivalently thoroughly studied in order to better describe their functions and their interaction with T cell subsets and neural cells in the CNS.

## Author Contributions

AW and TO designed the work. AW, KJ, AJ, JM, RK, and OC performed the experiments. AW, OC, and TO analyzed the data and wrote the paper. All authors read and approved the final manuscript.

## Conflict of Interest Statement

The authors declare that the research was conducted in the absence of any commercial or financial relationships that could be construed as a potential conflict of interest.

## Funding

This work has been supported by grants to TO from Lundbeckfonden (2012-12307, 2014-717), NovoNordiskFonden (NNF13OC0006131), Scleroseforeningen (R399-A27689), and Region of Southern Denmark Health Research.
